# Traducción y adaptación transcultural al contexto español del *Person-Centred Practice Inventory-Staff (PCPI-S)* para profesionales de la salud

**DOI:** 10.23938/ASSN.1039

**Published:** 2023-08-16

**Authors:** Begoña Errasti-Ibarrondo, Virginia La Rosa-Salas, Marta Lizarbe-Chocarro, Yvonne Gavela-Ramos, Ana Choperena, Leire Arbea Moreno, Mónica Vázquez-Calatayud, María José Galán-Espinilla, Brendan McCormack, Ana Carvajal-Valcárcel

**Affiliations:** 1 Universidad de Navarra Departamento de Enfermería de la Persona Adulta Facultad de Enfermería Universidad de Navarra Pamplona Spain; 2 Universidad de Navarra Grupo de investigación ICCP-UNAV, Innovación para un Cuidado Centrado en la Persona Universidad de Navarra Pamplona Spain; 3 Universidad de Navarra Instituto de Investigación Sanitaria de Navarra (IdiSNA) Pamplona Spain; 4 Universidad de Navarra Unidad de Docencia Práctica Facultad de Enfermería Universidad de Navarra Pamplona Spain; 5 Universidad de Navarra Instituto de Lengua y Cultura Españolas (ILCE) Facultad de Filosofía y Letras Universidad de Navarra Pamplona Spain; 6 Departamento de Oncología Radioterápica Clínica Universidad de Navarra Pamplona España; 7 Área de Desarrollo Profesional e Investigación en Enfermería Clínica Universidad de Navarra Pamplona España; 8 Servicio Navarro de Salud-Osasunbidea Centro de Salud de Ultzama Gerencia de Atención Primaria de Navarra Servicio Navarro de Salud-Osaunbidea Pamplona Spain; 9 The Susan Wakil School of Nursing and Midwifery. The University of Sydney. Sydney. Australia. University of Sydney The Susan Wakil School of Nursing and Midwifery The University of Sydney Sydney Australia

**Keywords:** Adaptación transcultural, Práctica centrada en la persona, Cuidado centrado en la persona, Profesionales de la salud, Encuestas y cuestionarios, Cross-cultural adaptation, Person-Centered practice, Person-centered care, Healthcare provider, Surveys and questionnaires

## Abstract

**Fundamento:**

El Cuidado Centrado en la Persona (CCP) se ha convertido en un tema central dentro del ámbito sanitario acorde con las políticas de salud nacionales e internacionales. El *Person Centred Practice Inventory Staff (PCPI-S)* es un instrumento basado en el modelo teórico *Person-Centred Practice Framework* que evalúa la percepción que tienen los profesionales de la salud sobre una práctica centrada en la persona. El objetivo del estudio es obtener la primera versión española del PCPI-S traducido y adaptado a nuestro contexto español.

**Método:**

Se llevó a cabo una traducción y adaptación cultural del instrumento utilizando la guía *Translation and Cultural Adaptation of Patient Reported Outcomes Measures - Principles of Good Practice* (PGP) que incluyó una sesión con expertos. También se realizó una validación de contenido de la claridad y relevancia de cada ítem (I-CVI), así como del cuestionario total (S-CVI/Ave).

**Resultados:**

No se encontraron dificultades para llegar a un consenso en los doce ítems que necesitaron ser clarificados. El índice de validez de contenido por ítem (I-CVI) obtuvo una puntuación excelente para claridad en 53 ítems, y para relevancia en 59; el índice de validez de contenido del cuestionario (S-CVI/Ave) mostró resultados excelentes (≥90).

**Conclusiones:.:**

Se ha obtenido la primera versión del PCPI-S adaptada al español, conceptual y semánticamente equivalente al cuestionario original. Este instrumento permitirá identificar la percepción que tienen los profesionales de la salud sobre una práctica centrada en la persona.

## Abstract (en)

**Background:**

Person-centred practices - following national and international developments in health-care policies - have become a key approach in healthcare. The Person-Centred Practice Inventory - Staff is an instrument based on the theoretical framework Person-Centred Practice that focuses on the staff’s perspective and how they experience person-centred practices. Here, the aim of this study is to obtain the first Spanish version of the PCPI-S translated and adapted into the Spanish context.

**Methods:**

The translation and adaptation of the instrument followed the Translation and Cultural Adaptation of Patient Reported Outcomes Measures *-* Principles of Good Practice, which included a consulting session with experts. Content validation measures on clarity and relevance were assessed for every item (I-CVI) and the survey as a whole (S-CVI/Ave).

**Results:**

No major difficulties were registered to reach an agreement on the 12 items that needed to be clarified. Regarding clarity and relevance. The validity index per item (I-CVI) obtained excellent scores for clarity in 53 items and for relevance in 59; the S-CVI/Ave showed excellent results (≥90).

**Conclusions:**

This first version of the Person-Centred Practice Inventory - Staff instrument adapted to the Spanish context is conceptually and semantically equivalent to the original one. This valuable tool will be of great help to identify the perception of healthcare professionals on person-centred practices.

## INTRODUCCIÓN

El Cuidado Centrado en la Persona (CCP) es un enfoque de cuidado holístico que considera a la persona en su totalidad como un individuo único y singular, y no la reduce a su enfermedad o a sus síntomas^1^^,^^2^. Al contemplar a la persona bajo esta perspectiva, los profesionales de la salud llegan a conocer y comprender su historia de vida, sus experiencias de salud, sus preferencias, qué es importante para ella y el papel que desempeñan su familia o las personas importantes en su vida^2^. Este enfoque implica cuidar a la persona con respeto, dignidad y compasión, proporcionándole apoyo y un tratamiento personalizado y coordinado^1^. Para ello, es fundamental que se establezcan relaciones terapéuticas entre los profesionales de la salud, los pacientes y sus personas más cercanas^3^^,^^4^, proporcionando los cuidados de salud dentro de una relación recíproca e igualitaria entre los profesionales, los pacientes y los familiares. Para que el cuidado sea el más adecuado posible, la información, la toma de decisiones y la prestación de los servicios han de responder en todo momento a las necesidades que manifiestan las personas^5^^,^^6^.

Los beneficios que se derivan de un sistema sanitario que promueve el enfoque de un CCP son numerosos para las partes implicadas. Para el *paciente*: mejor acceso al cuidado, aumentar los conocimientos de salud, facilitar la toma de decisiones informadas y compartidas en relación al tratamiento/cuidado, aumentar la adherencia a los tratamientos/cuidados, mejorar el manejo de la salud y el autocuidado^5^, mejorar los resultados clínicos y de salud^6^^,^^7^, y aumentar la satisfacción de los pacientes con la atención recibida. Para los *profesionales*: incrementar la satisfacción laboral y reducir las quejas recibidas por malas prácticas o por proporcionar una atención inadecuada^6^. Para el *sistema sanitario* en general: mejorar la eficiencia de los servicios, mejorar la continuidad de los entornos de atención primaria, atención hospitalizada, rehabilitación, largo término, paliativos, etc. y, de forma global, reducir los costes de la atención sanitaria^6^^,^^8^. Cabe recordar que la Organización Mundial de la Salud apostó por este enfoque en 2016^6^^,^^9^^,^^10^ como un marco fundamental para lograr una atención sanitaria de alta calidad que fomenta no solo la humanización de los servicios sanitarios, sino también la mejora de los resultados de salud, la coste-efectividad de los servicios y la calidad del propio cuidado^11^^,^^12^.

Esta visión está influyendo en todas las profesiones en las que el cuidado resulta un elemento nuclear^3^, como demuestra la diversidad de modelos y marcos sanitarios emergentes que se están empleando para guiar el desarrollo de la práctica, de las organizaciones y de las estrategias sanitarias centradas en la persona, y la variedad de términos que dan nombre a esos modelos y marcos, así como a sus elementos clave^3^^,^^9^. En efecto, hoy en día resulta imprescindible responder a las necesidades del individuo desde un enfoque de CCP tanto desde el punto de vista global de la humanización del cuidado, como de la eficiencia y la efectividad de los servicios sanitarios^13^.

Evaluar los resultados de una práctica centrada en la persona resulta complejo^9^. Una estrategia útil para determinar su implementación en la práctica es explorar las percepciones que tienen los profesionales de la salud y los propios pacientes sobre el CCP^10^^,^^14^.

No son abundantes los instrumentos que miden cómo los profesionales perciben el CCP^1^^,^^5^, y son escasos los que se basan en marcos y se dirigen a profesionales de distintos ámbitos sanitarios^5^. Por ejemplo, el *Person-Centred Care Assessment Tool* (PCAT)^15^^,^^16^ y el *Person-Directed Care* (PDC)^17^, ambos traducidos y validados al idioma español, no están fundamentados en un marco teórico y están dirigidos de manera exclusiva a los profesionales que cuidan a personas ancianas, y el recientemente desarrollado y validado *Person-Centered Care-Gerontology-Staff* (PCC-G-Staff) se basa en un modelo de CCP no teórico y su aplicación se centra en la población anciana^18^.

Por el contrario, el instrumento *Person-Centred Practice Inventory-Staff* (PCPI-S), traducido, adaptado y validado en diferentes idiomas y con buenas características psicométricas^9^^,^^10^^,^^19^^-^^21^, mide la percepción del CCP que tienen los profesionales de la salud en cualquier ámbito sanitario y se basa en el marco teórico internacionalmente reconocido *Person-Centred Practice Framework* (PCPF), desarrollado por McCormack y McCance^3^ y que ha sido recientemente traducido y adaptado al contexto español^22^ (Anexo I)^23^.

En virtud de esta escasez, y por todo lo anteriormente descrito, el objetivo de este estudio es obtener la primera versión española del instrumento *Person-Centred Practice Inventory-Staff* (PCPI-S) traducida y adaptada al contexto español.

## MÉTODO

### Diseño

Se desarrolló un estudio de adaptación transcultural, que consiste en obtener la equivalencia conceptual, semántica y de contenido del cuestionario^24^ *Person-Centred Practice Inventory-Staff* (PCPI-S). Para ello se siguieron los pasos del marco *Translation and Cultural Adaptation of Patient Reported Outcomes Measures - Principles of Good Practice* (PGP), diseñado por el grupo internacional *Translation and Cultural Adaptation group of International Society for Pharmacoeconomics and Outcome Research’s Quality of Life Special interest Group*^25^.

### Participantes

Las características de las personas involucradas en las distintas fases del proceso de adaptación transcultural se muestran en la Tabla 1.

**Tabla 1:** Participantes y sus roles en la adaptación transcultural del cuestionario *Person-Centred Practice Inventory-Staff* (PCPI-S)

Persona responsable	Fases	Formación y experiencia
*Autor del cuestionario*		
BMcC	1,5,6,	Experto en el CCP.
*Coordinadora del proyecto y Persona de contacto en nuestro contexto español*		
ACV	1,3,5,6,8,10	Nativa española.
		Nivel fluido de inglés.
		Residente en España.
		Ámbito de la salud.
		Familiarizada con el CCP.
		Experta en validaciones psicométricas.
		Experiencia en traducción de cuestionarios.
*Personas nativas españolas que actuaron como traductoras*		
BEI	2,3,5,8	Nivel fluido de inglés.
		Familiarizada con el CCP.
YGR	2,3,5,8	Nivel fluido de inglés.
		Traductora profesional.
		No familiarizada con ámbito sanitario ni CCP.
*Investigadora nativa española del comité de conciliación*		
VLS	3,5,8	Nivel fluido de inglés.
*Traductores profesionales nativos ingleses*		
CO	4	Nivel fluido en castellano.; No familiarizado con ámbito sanitario ni CCP.
AH		
*Panel de expertos*		
MVC	7	Enfermera con grado de doctor.
		Ámbito de gestión sanitaria.
		Familiarizada con el CCP.
MGE		Enfermera con grado de máster.
		Ámbito de gestión sanitaria.
LAM		Médico con grado de doctor.
		Ámbito clínico-académico.
MLCh		Enfermera con grado de doctor.
		Ámbito clínico-académico.
		Familiarizada con el CCP.
		Experta en validaciones psicométricas.
JMM		Enfermera con grado de doctor.
		Ámbito académico.
		Familiarizada con el CCP.
MOL		Enfermera con grado de doctor.
		Ámbito académico.
		Familiarizada con el CCP.
*Experta en humanidades*		
AChA	9	Familiarizada con CCP.
		Experiencia en correcciones gramaticales y lingüísticas.

CCP: cuidado centrado en la persona.

### Instrumento de medida

Como se ha mencionado previamente, el PCPI-S es un instrumento diseñado para medir la percepción que tienen los profesionales de la salud del CCP en cualquier ámbito sanitario, basado en el marco teórico PCPF^10^ (Anexo I). Está validado en Noruega^9^ (alfa de Chronbach (α): 0,88-0,93), Reino Unido^10^ (carga factorial: 0,41-0,92), Alemania^19^ (α: 0,90-0,94), Malasia^20^ (α: 0,59-0,86) y Corea^21^ (α =0.95).

Consta de 59 ítems agrupados en 17 constructos que pertenecen a los tres primeros dominios del PCPF (Tabla 2). Valora la percepción de los profesionales en una escala tipo Likert de 1 a 4: 1= totalmente en desacuerdo, 2= en desacuerdo, 3= neutral, 4= de acuerdo y 5= totalmente de acuerdo. La puntuación numérica proporcionada no posee la intención de cuantificar el grado de CCP que el profesional aporta, sino de orientar sobre cómo el profesional percibe un CCP.

**Tabla 2:** Relación entre los dominios y constructos del marco teórico *Person-Centred Practice Framework* (PCPF) y el número de ítems del cuestionario *Person-Centred Practice Inventory-Staff* (PCPI-S)

Dominios		Ítems (n=59)
*Prerrequisitos (requisitos necesarios)*		*18*
Constructos (n=5)	Ser profesionalmente competente	3
	Haber desarrollado habilidades interpersonales	4
	Estar comprometido con el trabajo	5
	Conocerse a sí mismo	3
	Claridad en las creencias y valores	3
*El entorno de la práctica*		*25*
Constructos (n=7)	Proporción adecuada de profesionales capacitados	3
	Sistemas de toma de decisiones compartida	4
	Relaciones eficaces entre profesionales	3
	Poder compartido	4
	Capacidad de innovación y asunción de riesgos	3
	El entorno físico	3
	Sistemas organizativos de apoyo	5
*Procesos centrados en la persona*		*16*
Constructos (n=5)	Trabajo con las creencias y los valores de la persona	4
	Toma de decisiones compartida	3
	Implicación auténtica	3
	Estar presente con pleno reconocimiento del otro	3
	Trabajar de manera holística	3

### Procedimiento

Se siguieron los diez pasos del marco *Translation and Cultural Adaptation of Patient Reported Outcomes Measures - Principles of Good Practice* (PGP) que se detallan a continuación:

1. *Preparación*: la coordinadora del proyecto contactó con uno de los autores del instrumento.
2. *Traducción*: las dos traductoras españolas tradujeron el instrumento del inglés al español de forma independiente, obteniendo las versiones VE_1_ y VE_2_.
3. *Conciliación*: un comité (las dos traductoras de la fase anterior, una investigadora y la coordinadora del proyecto) analizó y discutió las ambigüedades y diferencias de cada ítem, obteniendo por consenso la nueva versión española del instrumento (VE_3_).
4. *Retro traducción*: los dos traductores profesionales ingleses tradujeron la nueva versión española consensuada del instrumento (VE_3_) al inglés de manera independiente, obteniendo las versiones VI_1_ y VI_2_.
5. *Revisión de la retro traducción*: el comité de la fase de Conciliación consensuó la nueva versión inglesa del cuestionario (VI_3_) tras comparar las versiones VI_1_ y VI_2_. Esta versión fue revisada y comparada con la versión original por el autor del cuestionario. Una vez finalizada esta fase se preguntó al comité por su percepción en el proceso de traducción.
6. *Armonización*: el autor del cuestionario comparó la retro traducción inglesa de la versión noruega^6^ con la retrotraducción inglesa de la versión española (VI_3)_ para valorar si había discrepancias entre sí.
7. *Sesión cognitiva*: el panel de seis expertas cumplimentó el cuestionario. Se calculó el tiempo requerido para completarlo y se evaluó la claridad y relevancia de cada ítem mediante una escala Likert de cuatro puntos definiendo como 1= no relevante/no claro hasta 4=muy relevante/muy claro^26^. Además, para los ítems con puntuaciones de 1 o 2 en relevancia o claridad, se solicitó que propusieran términos alternativos. Posteriormente, se realizó una puesta en común para especificar los motivos de esas puntuaciones y justificar las discrepancias encontradas. Finalmente, el instrumento fue testado en un grupo de cinco profesionales sanitarios, población a la que va dirigida el instrumento. Se midió también el tiempo requerido para cumplimentar el cuestionario y su percepción a la hora de completarlo.
8. *Revisión de los resultados de la sesión*: las dos investigadoras del proyecto expertas en psicometría calcularon el índice de validez de contenido por ítem (I-CVI) y el índice de validez de contenido de todo el cuestionario (S-CVI/Ave). Posteriormente, aquellos aspectos que necesitaron ser clarificados fueron consultados al autor del cuestionario. Seguidamente, se analizaron los comentarios de los expertos y la población diana.
9. *Corrección gramatical de la nueva versión*: la investigadora experta en humanidades corrigió los errores léxico-semánticos (palabras), morfosintácticos (concordancia gramatical de género y número, modo y tiempo verbal) y ortográficos de la última versión del cuestionario, que fueron consensuados con la coordinadora del proyecto.
10. *Informe final*: la coordinadora del proyecto escribió el informe final incluyendo los participantes, las metodologías utilizadas y las decisiones tomadas en todo el proceso.

### Análisis de datos

En una primera fase cuantitativa se midió el índice de validez de contenido por ítem (I-CVI) y el índice de validez de contenido de todo el cuestionario (S-CVI/Ave). La claridad y relevancia de cada ítem (I-CVI) se obtuvo dividiendo el número de observadores que puntuaron los ítems con 3 o 4 entre el total de observadores; se calculó el índice Kappa modificado^26^ para evitar la posibilidad de coincidencia debida al azar entre los expertos, y los resultados se interpretaron según la clasificación de Cicchetti y Sparrow^27^. La S-CVI/Ave se calculó como el promedio de los I-CVI, interpretándose como excelente si el valor obtenido era superior a 0,90^28^. En la segunda fase, cualitativa, se analizó el contenido de los comentarios sugeridos por los expertos y por la población diana.

## RESULTADOS

Se obtuvieron los siguientes resultados correspondientes a las fases seguidas para el proceso de traducción y adaptación transcultural del PCPI-S:

1. ***Preparación***
  Se obtuvo el consentimiento del autor del cuestionario para participar en el proceso.
2. ***Traducción***
  Se obtuvieron las versiones en español VE_1_ y VE_2_; los traductores comentaron que no revistió complicación a pesar de ser un instrumento extenso (necesitaron dos horas para traducirlo).
3. ***Conciliación***
  El comité obtuvo por consenso la versión en español VE_3_, con las siguientes matizaciones:
  - Ítems 11 y 34: se vio la necesidad de clarificar el término “*evidence”* añadiendo entre paréntesis una explicación de su significado (*experiencias de otros profesionales, opiniones de los demás, preferencias de pacientes y familias, etc*.).
  - Ítem 16: no se encontró un término apropiado para traducir el vocablo “*feedback”*, por lo que se decidió mantenerlo en el inglés original.
  - Ítem 20: se acordó clarificar el enunciándolo de la siguiente manera: *soy capaz de plantear y justificar aquellas situaciones en las que los conocimientos y habilidades no alcanzan niveles aceptables*.
  - Todo el cuestionario: se decidió utilizar la palabra “*cuidado*” (*care*) porque se considera que todos los profesionales de salud cuidan.
4. ***Retro-traducción***
  Las versiones en inglés VI_1_ y VI_2_ se obtuvieron de manera conceptual en vez de literal; los traductores comentaron que no tuvieron problemas para traducir el instrumento.
5. ***Revisión de la retro-traducción***
  Se obtuvo la versión inglesa consensuada VI_3_. El autor del cuestionario sugirió modificar doce ítems de la versión española (1, 9, 11, 15, 16, 17, 22, 31, 37, 46, 49 y 53), cuyas modificaciones se muestran en la Tabla 3. El comité consideró que, a pesar de ser un instrumento extenso (59 ítems), las preguntas estaban muy relacionadas entre sí y la traducción fue fácil de realizar. Ante las discrepancias fue fácil llegar a un consenso porque el comité estaba familiarizado con el CCP.
  **Tabla 3:** Versión consensuada en inglés obtenida tras los comentarios del autor del cuestionario en la fase 5

Ítems	Versión inglesa original	Retro traducción del español al inglés (VI_3_)	Versión definitiva tras incluir las aportaciones del autor del cuestionario
1	I have the necessary skills to negotiate care options	I have the necessary skills to reach an agreement on care options.	Tengo la preparación necesaria para consensuar las opciones de cuidado
9	I seek opportunities to get to know the person and their family in order to provide holistic care	I endeavour to get to know the people and their families in order to provide holistic care.	Me preocupo por conocer a la persona y a su familia para proporcionar un cuidado holístico
11	I strive to deliver high quality care that is informed by evidence	I strive to provide evidence-based high-quality care.	Me esfuerzo por proporcionar un cuidado de alta calidad basado en distintas evidencias (experiencia del profesional, opiniones de los demás y preferencias del paciente y su familia, etc.)
15	I pay attention to how my life experiences influence my practice	I am aware of how my own experiences shape my clinical practice.	Soy consciente de cómo mis propias experiencias influyen en mi práctica
16	I actively seek feedback from others about my practice	I actively look for feedback from others about my clinical practice.	Busco de manera activa el *feedback* de los demás sobre mi práctica
17	I challenge colleagues when their practice is inconsistent with our team’s shared values and beliefs	I tell other professionals when their practice is not coherent with the team’s values and beliefs.	Hago ver a otros profesionales las situaciones en las que su práctica no se corresponde con los valores y creencias del equipo
22	I actively participate in team meetings to inform my decision-making	I actively participate in team meetings for decision-making.	Participo activamente en las reuniones de equipo para que pueda tomar decisiones.
31	The leader facilitates participation	The team leader facilitates participation.	La/el líder favorece la participación
37	I challenge others to consider how different elements of the physical environment impact on person-centredness (e.g. noise, light, heat etc)	I point out to others how certain aspects of the physical environment may have an impact on person-centred care (e.g. noise, light, heat, etc.).	Hago que otros se planteen cómo algunos elementos del entorno físico influyen en el cuidado centrado en la persona (ej. ruidos, luz, calor, etc.).
46	I seek feedback on how people make sense of their care experience	I seek feedback on how people.	Busco *feedback* sobre cómo las personas viven y le dan sentido a su cuidado.
49	I work with the person to set health goals for their future	I work with the person to set long-term health goals.	Trabajo con la persona para establecer objetivos de salud de cara a su futuro.
53	I engage people in care processes where appropriate	I involve people in their healthcare process whenever appropriate.	Implico a las personas en su proceso asistencial cuando es adecuado.
6. ***Armonización***
  El autor del cuestionario no encontró diferencias significativas entre las diferentes versiones.
7. ***Sesión cognitiva***
  En la puesta en común se discutieron los siguientes ítems.
  - ítems 8 y 11: se propuso unificarlos pero no se unificaron porque el autor del cuestionario clarificó que el ítem 11 “*me esfuerzo por proporcionar un cuidado de alta calidad basado en distintas evidencias (experiencia de otros profesionales, opiniones de los demás, preferencias del paciente y su familia, etc*.)” es más específico y complementa al ítem 8 “*me esfuerzo por proporcionar un cuidado de alta calidad”*.
  - ítem 9: se discutió la posibilidad de modificar el término “*cuidado holístico* para que lo entendieran todos los profesionales de la salud; sin embargo, se mantuvo porque los expertos estuvieron de acuerdo en que se trata de un término ampliamente utilizado en el ámbito sanitario.
  - ítems 13 y 14: se propuso unificarlos pero se acordó no hacerlo, ya que abordan aspectos distintos: el ítem 13 se centra en el tiempo dedicado a reflexionar sobre las reacciones (“*dedico tiempo a reflexionar sobre mis reacciones en situaciones determinadas*”) y en el ítem 14 se valora la reflexión sobre las acciones (“*reflexiono sobre si mis acciones son coherentes con mi modo de ser*”).
  - ítem 18: se cambió el término “*favorezco*” por el término “*apoyo*”.
  - ítems 26 y 27: se propuso unificarlos aunque finalmente se acordó mantenerlos ambos ya que, aunque los dos valoran el trabajo en equipo, el ítem 26 se centra en valorar la contribución al equipo (“*trabajo en un equipo en el que se valora mi contribución para proporcionar un cuidado centrado en la persona*”) y el ítem 27 se focaliza en fomentar que los miembros contribuyan a trabajar en equipo (“*trabajo en un equipo en el que se fomenta que todos los miembros contribuyan al cuidado centrado en la persona*”).
  - ítem 31: se planteó sustituir el término “*líder*” por “*jefes*” pero, tras consultarlo con el autor del cuestionario, se decidió no modificarlo porque el término “*líder*” se refiere a cualquier profesional y no solo a jefes o directivos.
  - ítem 44: se sugirió cambiar el término “*integro*” por “*utilizo*”; sin embargo, se vio que no tenía el mismo significado y se mantuvo el término original.
  - ítem 45: se cambiaron los términos “*de su familia y sus cuidadores”* por *“de su familia y el de sus cuidadores*”.
  - ítem 47: se cambió “*animo a las personas a que compartan lo que es importante para ellas*” por “*animo a que las personas compartan lo que es importante para ellas*”.
  - ítem 52: se añadió el adjetivo “*cuidada*” al sustantivo “*persona*”, resultando el término “*persona cuidada*”.
  - ítem 54: se modificaron los términos “*a las personas que reciben cuidados*” por “*a las personas que cuido*”.
  Respecto a la percepción de los cinco profesionales que cumplimentaron el cuestionario en la prueba piloto, todos consideraron que el instrumento se entendía bien y cubría aspectos relacionados con su práctica clínica diaria.
8. ***Revisión de los resultados***
  *Claridad*: La mayoría de los ítems obtuvieron una puntuación de I-CVI clasificada como *excelente*, a excepción de los que obtuvieron una puntuación *aceptable* (4, 12, 29, 34, 53 y 57). El índice de validez de contenido de todo el cuestionario también se clasificó como excelente (S-CVI/Ave = 0,90) (Tabla 3).
  *Relevancia*: Todos los ítems obtuvieron una puntuación de I-CVI clasificada como *excelente*; S-CVI/Ave fue 0,96 (Tabla 4).
9. ***Corrección gramatical del cuestionario***
  Se corrigieron los errores en cinco ítems (12, 35, 38, 44 y 46).
10. ***Informe final***
  La Tabla 5 incluye la versión definitiva del cuestionario.
  **Tabla 4:** Claridad y relevancia de la versión española del cuestionario *Person-Centred Practice-Inventory-Staff* (PCPI-S)

		Claridad			Relevancia		
Item	Constructo	I-CVI	K		I-CVI	K	
	*Ser profesionalmente competente*						
1	Tengo la preparación necesaria para consensuar opciones de cuidado	1	1	E	1	1	E
2	Cuando cuido, mi atención va más allá de la propia dimensión física	1	1	E	1	1	E
3	Busco oportunidades para ampliar mi competencia profesional de forma activa	0,83	0,81	E	0,83	0,81	E
	*Haber desarrollado habilidades interpersonales*						
4	Me aseguro de escuchar y reconocer las perspectivas de los demás	0,67	0,57	A	1	1	E
5	Cuando me comunico, muestro respeto hacia los demás	0,83	0,81	E	1	1	E
6	Utilizo diferentes estrategias de comunicación para llegar a soluciones de mutuo acuerdo	0,83	0,81	E	0,83	0,81	E
7	Soy consciente de cómo mi comunicación no verbal influye en el modo de relacionarme con los demás	1	1	E	1	1	E
	*Estar comprometido con el trabajo*						
8	Me esfuerzo por proporcionar un cuidado de alta calidad	0,83	0,81	E	0,67	0,57	A
9	Me preocupo por conocer a la persona y a su familia para proporcionar un cuidado holístico	0,83	0,81	E	1	1	E
10	Me esfuerzo por dedicar tiempo a las personas que cuido	1	1	E	1	1	E
11	Me esfuerzo por proporcionar un cuidado de alta calidad basado en distintas evidencias (experiencia de otros profesionales, opiniones de los demás, preferencias del paciente y su familia, etc.)	0,83	0,81	E	0,83	0,81	E
12	Continuamente busco oportunidades para mejorar la experiencia de cuidado de la persona	0,67	0,57	A	0,83	0,81	E
	*Conocerse a sí mismo*						
13	Dedico tiempo a reflexionar sobre mis reacciones en situaciones determinadas	1	1	E	1	1	E
14	Reflexiono sobre si mis acciones son coherentes con mi modo de ser	0,83	0,81	E	0,83	0,81	E
15	Soy consciente de cómo mis propias experiencias influyen en mi práctica	1	1	E	1	1	E
	*Claridad en las creencias y valores*						
16	Busco de manera activa el *feedback* de los demás sobre mi práctica	0,83	0,81	E	1	1	E
17	Hago ver a otros profesionales las situaciones en las que su práctica no se corresponde con los valores y creencias del equipo	1	1	E	1	1	E
18	Apoyo a otros profesionales para que desarrollen una práctica acorde con los valores y creencias del equipo	0,83	0,81	E	1	1	E
	*Proporción adecuada de profesionales capacitados*						
19	Detecto cuando hay carencias de conocimiento y habilidades en el equipo, y el impacto que esto tiene en su cuidado	1	1	E	1	1	E
20	Soy capaz de plantear y justificar aquellas situaciones en las que la ratio y el perfil de los profesionales no alcanzan un nivel aceptable	0,83	0,81	E	1	1	E
21	Valoro las aportaciones que hacen todos los miembros del equipo y su contribución al cuidado	1	1	E	1	1	E
	*Sistemas de toma de decisiones compartida*						
22	Participo activamente en las reuniones de equipo para fundamentar de forma adecuada mis decisiones	0,83	0,81	E	1	1	E
23	Participo en los foros de la institución en los que se toman decisiones que influyen en la práctica	1	1	E	1	1	E
24	Tengo oportunidades para participar de una forma activa en la toma de decisiones de mi unidad/ departamento	1	1	E	1	1	E
25	En las sesiones clínicas de toma de decisiones buscan mi opinión (ej. pases de visita, consulta de casos, planificación del alta)	1	1	E	1	1	E
	*Relaciones eficaces entre profesionales*						
26	Trabajo en un equipo en el que se valora mi contribución para proporcionar un cuidado centrado en la persona	1	1	E	1	1	E
27	Trabajo en un equipo en el que se fomenta que todos los miembros contribuyan al cuidado centrado en la persona	1	1	E	1	1	E
28	Mis colegas son un ejemplo a seguir para el desarrollo de relaciones eficaces	0,83	0,81	E	0,83	0,81	E
	*Poder compartido*						
29	Se reconoce y aprecia la contribución de los colegas	0,67	0,57	A	1	1	E
30	Contribuyo de manera activa al desarrollo de objetivos comunes	1	1	E	1	1	E
31	La/el líder favorece la participación.	1	1	E	1	1	E
32	Se me anima y apoya para poner en marcha cambios en la práctica	1	1	E	1	1	E
	*Capacidad de innovación y asunción de riesgos*						
33	Me siento apoyado/a para hacer las cosas de forma diferente con el fin de mejorar mi práctica	1	1	E	0,83	0,81	E
34	Soy capaz de sopesar el uso de la evidencia con los posibles riesgos en la toma de decisiones	0,67	0,57	A	0,83	0,81	E
35	Estoy comprometido/a con la mejora del cuidado cuestionando mi práctica	0,83	0,81	E	1	1	E
	*El entorno físico*						
36	Presto atención al impacto que tiene el entorno físico en la dignidad de las personas	1	1	E	1	1	E
37	Hago que otros se planteen cómo el entorno físico puede influir en el cuidado centrado en la persona (ej. ruidos, luz, calor, etc.)	1	1	E	1	1	E
38	Busco formas creativas de mejorar el entorno físico	1	1	E	0,83	0,81	E
	*Sistemas organizativos de apoyo*						
39	En el equipo dedicamos tiempo a celebrar los logros	1	1	E	1	1	E
40	Mi institución reconoce y recompensa los logros	1	1	E	1	1	E
41	Se me reconoce por fomentar que las personas tengan una experiencia muy positiva con el cuidado	1	1	E	0,83	0,81	E
42	Me siento apoyado/a para expresar mis inquietudes sobre cualquier aspecto del cuidado	1	1	E	0,83	0,81	E
43	Habitualmente tengo la oportunidad para hablar de mi práctica y desarrollo profesional	1	1	E	1	1	E
	*Trabajo con las creencias y los valores de la persona*						
44	Integro en el cuidado el conocimiento que tengo de la persona	0,83	0,81	E	1	1	E
45	Trabajo con la persona teniendo en cuenta el contexto de su familia y el de sus cuidadores	1	1	E	1	1	E
46	Busco *feedback* sobre cómo las personas le dan sentido a su cuidado	0,83	0,81	E	1	1	E
47	Animo a que las personas compartan lo que es importante para ellas	0,83	0,81	E	1	1	E
	*Toma de decisiones compartida*						
48	Incluyo a la familia en la toma de decisiones cuando es adecuado y de acuerdo con los deseos de la persona	0,83	0,81	E	1	1	E
49	Trabajo con la persona para establecer objetivos de salud de cara a su futuro	1	1	E	1	1	E
50	Facilito que las personas a las que cuido soliciten información sobre su cuidado a otros profesionales sanitarios	1	1	E	1	1	E
	*Implicación auténtica*						
51	Intento comprender la perspectiva de la persona	0,83	0,81	E	1	1	E
52	Intento llegar a un acuerdo cuando mis objetivos difieren de los de la persona cuidada	0,83	0,81	E	1	1	E
53	Implico a las personas en su proceso asistencial cuando es adecuado	0,67	0,57	A	1	1	E
	*Estar presente con pleno reconocimiento del otro*						
54	Escucho activamente a las personas que cuido para identificar sus necesidades no cubiertas	0,83	0,81	E	1	1	E
55	Recojo información complementaria que me ayude a apoyar a las personas que reciben atención sanitaria	0,83	0,81	E	1	1	E
56	Me aseguro de que mi atención está plenamente centrada en la persona cuando estoy con ella	1	1	E	1	1	E
	*Trabajar de manera holística*						
57	Me esfuerzo por entender a la persona en su totalidad	0,67	0,57	A	1	1	E
58	Valoro las necesidades de la persona teniendo en cuenta todos los aspectos de su vida	1	1	E	1	1	E
59	Proporciono un cuidado teniendo en cuenta a la persona en su totalidad	1	1	E	1	1	E
S-CVI/ave		0,90 (E)			0,96 (E)		

I-CVI: *Item-level content validity index*: (validez de contenido por ítem); K: *kappa* modificado ajustado por azar; Ev: evaluación de kappa ponderado según Cicchetti y Sparrow (1981) y Fleiss (1981); A: aceptable (0,40-0,59); E: excelente (≥0,75); S-CVI/Ave: *Scale-level content valididy/average* (validez de contenido total de la escala/promedio), valorado según Waltz y col (2005); E: excelente (≥0,90).
  **Tabla 5:** Versión definitiva de la versión española del cuestionario *Person-Centred Practice Inventory-Staff* (PCPI-S)

Ítem	Constructo
*Ser profesionalmente competente*	
Item 1	Tengo la preparación necesaria para consensuar opciones de cuidado
Item 2	Cuando cuido, mi atención va más allá de la propia dimensión física
Item 3	Busco oportunidades para ampliar mi competencia profesional de forma activa
*Haber desarrollado habilidades interpersonales*	
Item 4	Me aseguro de escuchar y reconocer las perspectivas de los demás
Item 5	Cuando me comunico, muestro respeto hacia los demás
Item 6	Utilizo diferentes estrategias de comunicación para llegar a soluciones de mutuo acuerdo
Item 7	Soy consciente de cómo mi comunicación no verbal influye en el modo de relacionarme con los demás
*Estar comprometido con el trabajo*	
Item 8	Me esfuerzo por proporcionar un cuidado de alta calidad
Item 9	Me preocupo por conocer a la persona y a su familia para proporcionar un cuidado holístico
Item 10	Me esfuerzo por dedicar tiempo a las personas que cuido
Item 11	Me esfuerzo por proporcionar un cuidado de alta calidad basado en distintas evidencias (experiencia de otros profesionales, opiniones de los demás, preferencias del paciente y su familia, etc.)
Item 12	Continuamente busco oportunidades para mejorar la experiencia de cuidado de la persona
*Conocerse a sí mismo*	
Item 13	Dedico tiempo a reflexionar sobre mis reacciones en situaciones determinadas
Item 14	Reflexiono sobre si mis acciones son coherentes con mi modo de ser
Item 15	Soy consciente de cómo mis propias experiencias influyen en mi práctica
*Claridad en las creencias y valores*	
Item 16	Busco de manera activa el *feedback* de los demás sobre mi práctica
Item 17	Hago ver a otros profesionales las situaciones en las que su práctica no se corresponde con los valores y creencias del equipo
Item 18	Apoyo a otros profesionales para que desarrollen una práctica acorde con los valores y creencias del equipo
*Proporción adecuada de profesionales capacitados*	
Item 19	Detecto cuando hay carencias de conocimiento y habilidades en el equipo, y el impacto que esto tiene en su cuidado
Item 20	Soy capaz de plantear y justificar aquellas situaciones en las que la ratio y el perfil de los profesionales no alcanzan un nivel aceptable
Item 21	Valoro las aportaciones que hacen todos los miembros del equipo y su contribución al cuidado
*Sistemas de toma de decisiones compartida*	
Item 22	Participo activamente en las reuniones de equipo para fundamentar de forma adecuada mis decisiones
Item 23	Participo en las foros de la institución en los que se toman decisiones que influyen en la práctica
Item 24	Tengo oportunidades para participar de una forma activa en la toma de decisiones de mi unidad/ departamento
Item 25	En las sesiones clínicas de toma de decisiones buscan mi opinión (ej. pases de visita, consulta de casos, planificación del alta)
*Relaciones eficaces entre profesionales*	
Item 26	Trabajo en un equipo en el que se valora mi contribución para proporcionar un cuidado centrado en la persona
Item 27	Trabajo en un equipo en el que se fomenta que todos los miembros contribuyan al cuidado centrado en la persona
Item 28	Mis colegas son un ejemplo a seguir para el desarrollo de relaciones eficaces
*Poder compartido*	
Item 29	Se reconoce y aprecia la contribución de los colegas
Item 30	Contribuyo de manera activa al desarrollo de objetivos comunes
Item 31	La/el líder favorece la participación.
Item 32	Se me anima y apoya para poner en marcha cambios en la práctica
*Capacidad de innovación y asunción de riesgos*	
Item 33	Me siento apoyado/a para hacer las cosas de forma diferente con el fin de mejorar mi práctica
Item 34	Soy capaz de sopesar el uso de la evidencia con los posibles riesgos en la toma de decisiones
Item 35	Estoy comprometido/a con la mejora del cuidado cuestionando mi práctica
*El entorno físico*	
Item 36	Presto atención al impacto que tiene el entorno físico en la dignidad de las personas
Item 37	Hago que otros se planteen cómo el entorno físico puede influir en el cuidado centrado en la persona (ej. ruidos, luz, calor, etc.)
Item 38	Busco formas creativas de mejorar el entorno físico
*Sistemas organizativos de apoyo*	
Item 39	En el equipo dedicamos tiempo a celebrar los logros
Item 40	Mi institución reconoce y recompensa los logros
Item 41	Se me reconoce por fomentar que las personas tengan una experiencia muy positiva con el cuidado
Item 42	Me siento apoyado/a para expresar mis inquietudes sobre cualquier aspecto del cuidado
Item 43	Habitualmente tengo la oportunidad para hablar de mi práctica y desarrollo profesional
*Trabajo con las creencias y los valores de la persona*	
Item 44	Integro en el cuidado el conocimiento que tengo de la persona
Item 45	Trabajo con la persona teniendo en cuenta el contexto de su familia y el de sus cuidadores
Item 46	Busco *feedback* sobre cómo las personas le dan sentido a su cuidado
Item 47	Animo a que las personas compartan lo que es importante para ellas
*Toma de decisiones compartida*	
Item 48	Incluyo a la familia en la toma de decisiones cuando es adecuado y de acuerdo con los deseos de la persona
Item 49	Trabajo con la persona para establecer objetivos de salud de cara a su futuro
Item 50	Facilito que las personas a las que cuido soliciten información sobre su cuidado a otros profesionales sanitarios
*Implicación auténtica*	
Item 51	Intento comprender la perspectiva de la persona
Item 52	Intento llegar a un acuerdo cuando mis objetivos difieren de los de la persona cuidada
Item 53	Implico a las personas en su proceso asistencial cuando es adecuado
*Estar presente con pleno reconocimiento del otro*	
Item 54	Escucho activamente a las personas que cuido para identificar sus necesidades no cubiertas
Item 55	Recojo información complementaria que me ayude a apoyar a las personas que reciben atención sanitaria
Item 56	Me aseguro de que mi atención está plenamente centrada en la persona cuando estoy con ella
*Trabajar de manera holística*	
Item 57	Me esfuerzo por entender a la persona en su totalidad
Item 58	Valoro las necesidades de la persona teniendo en cuenta todos los aspectos de su vida
Item 59	Proporciono un cuidado teniendo en cuenta a la persona en su totalidad

Piense y reflexione sobre su práctica diaria y, por favor, indique el grado de acuerdo o desacuerdo (1*: Totalmente en desacuerdo. 2: En desacuerdo. 3: Neutral. 4: De acuerdo. 5: Totalmente de acuerdo*) con respecto a las siguientes afirmaciones.

## DISCUSIÓN

Tras el proceso de traducción y adaptación transcultural del cuestionario PCPI-S se ha obtenido la primera versión española equivalente a la versión original a nivel semántico, conceptual y de contenido. Este es el primer paso para poder medir las propiedades psicométricas del instrumento y obtener la versión española válida y fiable, adaptada culturalmente al contexto español^29^.

El PCPI-S está fundamentado en el marco teórico PCPF^23^, también traducido y adaptado al español^22^. Dicho marco define los principales componentes para la implementación de un CCP garantizando que el PCPI-S mide la percepción de los distintos profesionales sobre las diferentes dimensiones que componen el CCP^10^^,^^30^. Además, la recurrencia de estudios que mencionan este instrumento desarrollado por McCormack y col^9^^,^^10^^,^^17^^,^^19^, especialmente en la literatura internacional, se puede relacionar con su eficacia y resultados positivos. La literatura destaca asimismo la carencia de instrumentos desarrollados bajo la fundamentación de marcos teóricos^15^. Por tanto, disponer de la versión española del PCPI-S llena este vacío encontrado en la evidencia.

El marco teórico PCPF^23^ también ha servido de base para el desarrollo de otros instrumentos como el *Person-Centred Practice Inventory-Care* (PCPI-C)^31^ y el *Person-Centred Practice Inventory-Student* (PCPI-ST)^32^ que evalúan la percepción de un CCP desde la perspectiva de pacientes y de estudiantes, respectivamente. Disponer de estos tres instrumentos (PCPI-C y PCPI-ST se encuentran en proceso de publicación) basados en un mismo marco conceptual, ayudará a garantizar la validez de la medición del mismo fenómeno, tanto desde el punto de vista de los agentes que proporcionan el cuidado (profesionales y estudiantes), como de aquellos que lo reciben (pacientes). Dado que los pacientes son los destinatarios del cuidado, resulta crucial identificar la percepción que los propios profesionales tienen del fenómeno CCP^33^ y hasta qué punto comparten la misma comprensión que los pacientes^34^. A este respecto, existen estudios que han revelado algunas diferencias entre las percepciones de las enfermeras y de los pacientes acerca de lo que podría denominarse un cuidado de enfermería *de calidad* o *un buen cuidado*^34^^,^^35^.

Además, este instrumento ha sido desarrollado para ser utilizado por diferentes agentes que proporcionan el cuidado (personal médico, enfermero, técnico de cuidados auxiliares de enfermería, etc.). El conocimiento de las distintas percepciones pone de manifiesto que el CCP precisa de un abordaje interprofesional y no de la mirada aislada de un único profesional^36^. Por ello, este enfoque complementa y completa los resultados de otros estudios que solamente se centran en conocer la percepción de un grupo de profesionales^37^.

Se puede destacar que la guía empleada para llevar a cabo el proceso de traducción y adaptación cultural en el presente estudio (*Translation and cultural adaptation process for Patient-Reported Outcomes (PRO) Principles of Good Practice (PGP)*^25^) es ampliamente utilizada y reconocida internacionalmente. Esta herramienta ya se ha aplicado para realizar el proceso de traducción y adaptación del PCPI-S al noruego^9^ y alemán^19^. La utilización de la misma guía en las tres adaptaciones lingüísticas del PCPI-S da consistencia a las distintas versiones obtenidas, asegurando la validez de la medición al ser aplicada a poblaciones de distintas culturas^38^.

Otra fortaleza del estudio es que el autor del cuestionario participó en todo el proceso de traducción y adaptación cultural. La literatura pone de manifiesto que la inclusión del autor del cuestionario es uno de los requisitos más importantes para alcanzar la máxima calidad durante el proceso^25^. Además, el autor del cuestionario es también el autor del marco teórico que sustenta el instrumento y por ello, es el experto clave de los conceptos y del significado de los ítems del PCPI-S. Esto ha permitido clarificar cuestiones sobre el instrumento en cualquier momento del proceso y asegurar el significado conceptual y no literal de los ítems.

Al igual que en las traducciones y adaptaciones del PCPI-S a otras culturas^9^^,^^19^^-^^21^, en el presente estudio tampoco se encontraron grandes dificultades en la traducción y adaptación del cuestionario al español.

Cabe resaltar que el término “cuidado”, clave en la atención centrada en la persona, aparece con frecuencia a lo largo de todo el cuestionario. Aunque este término se asocia principalmente al ámbito de la enfermería, y en la traducción y adaptación cultural del PCPI-S al contexto noruego^9^ se comprobó que este concepto no es aplicable a otras profesiones de la salud como la medicina o la psicología, en el presente estudio los distintos profesionales sanitarios del panel de expertos concluyeron que todos ellos cuidan, y que en nuestra cultura este término es generalizable y trasladable a todos los profesionales de la salud. Por ello, al igual que en la versión original del cuestionario, en la versión española se mantuvo el término “cuidado”. Sin embargo, es importante destacar que el cuidado es el elemento central de la disciplina enfermera^39^.

Respecto a la validación del instrumento, la validez de contenido mostró que el 100% de los ítems fueron congruentes con los constructos a medir^40^. Aunque la validación de contenido (realizada en la sesión cognitiva de la fase 7) fue realizada por un panel de cinco expertos de carácter interprofesional^41^, sería interesante que en futuros estudios se ampliase el número y el tipo de profesionales; por ejemplo, podrían incorporarse profesionales del área de Psicología.

En cuanto a las limitaciones, es importante tener en cuenta que este estudio se centra en la primera fase del proceso de validación y que es imprescindible realizar la medición de las propiedades psicométricas de validez y fiabilidad del instrumento, fase en la cual se encuentra inmerso actualmente el equipo investigador. Por tanto, este estudio garantiza la adaptación del PCPI-S a nuestro lenguaje y contexto cultural español, pero sin perder de vista la necesidad de volver a adaptarlo a otro contexto de habla hispana diferente al de nuestro país^42^.

Como conclusión, este estudio ha obtenido la primera versión traducida y adaptada al español del PCPI-S, conceptual y semánticamente equivalente al instrumento original. El seguimiento de una guía internacionalmente reconocida y ampliamente utilizada garantiza la rigurosidad del proceso y la calidad de la versión española obtenida.

Una vez se complete el proceso de validación con la medición de las propiedades psicométricas de validez y fiabilidad, se dispondrá de un instrumento para medir el cambio de percepciones de los profesionales acerca de una atención centrada en la persona tras el diseño e implementación de intervenciones orientadas a mejorar el cuidado con dicho enfoque, que garantizará la calidad de su utilización en el ámbito de la práctica clínica, de la docencia y de la investigación.

## Figures and Tables

**Figura 1:**
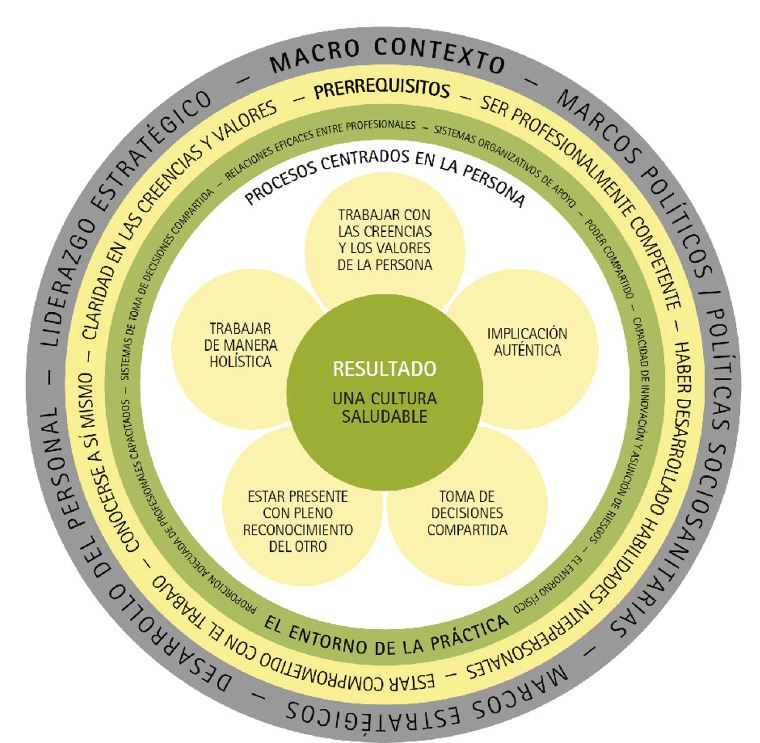
Versión traducida y adaptada al español del *Person Centred Practice Framework* (PCPF)*.

* Tomado de Choperena et al. 2022. Traducción y adaptación transcultural al contexto español del marco teórico *Person-Centred Practice Framework*. An Sist Sanit Navar 2022; 45(3): e1016. https://doi.org/10.23938/ASSN.1016. Se dispone de permiso de reproducción.

## ANEXO I Marco *Person Centred Practice Framework*

El marco *Person Centred Practice Framework* (PCPF) nació con el objetivo de operativizar una práctica centrada en la persona^3^, ^10^ y ofrecer a los profesionales de la salud un lenguaje que les permitiera abordar sus componentes y las barreras/facilitadores que influyen en su desarrollo^3^. El PCPF es multidisciplinar y aplicable en diferentes contextos, y consta de cinco dominios:

1. los prerrequisitos, que se centran en los atributos de los trabajadores;
2. *el entorno de la práctica*, que refleja la complejidad del contexto en el que se proporciona el cuidado;
3. *los procesos centrados en la persona*, que se entienden como las especificaciones -en cuanto al tipo de presencia/cuidado/relación- necesarias para interactuar con la persona;
4. *el resultado,* que deriva de una práctica centrada en la persona efectiva y que consiste en llegar a una cultura saludable;
5. *el macrocontexto,* que contempla los factores que influyen en el desarrollo de culturas centradas en la persona, y que son estratégicos y políticos por naturaleza^3^

Para llegar al resultado (una cultura saludable), primero se han de considerar los atributos de los trabajadores como prerrequisitos para manejar el entorno de la práctica y para comprometerse de forma efectiva en los procesos centrados en la persona (Fig. 1).
